# A Proteomic Screen for Nucleolar SUMO Targets Shows SUMOylation Modulates the Function of Nop5/Nop58

**DOI:** 10.1016/j.molcel.2010.07.025

**Published:** 2010-08-27

**Authors:** Belinda J. Westman, Céline Verheggen, Saskia Hutten, Yun Wah Lam, Edouard Bertrand, Angus I. Lamond

**Affiliations:** 1 Wellcome Trust Centre for Gene Regulation and Expression, College of Life Sciences, University of Dundee, Dundee DD15EH, UK; 2 Institut de Génétique Moléculaire de Montpellier, CNRS-UMR 5535, 34293 Montpellier, France; 3 Department of Biology and Chemistry, City University of Hong Kong, Kowloon, Hong Kong

**Keywords:** PROTEINS, RNA

## Abstract

Posttranslational SUMO modification is an important mechanism of regulating protein function, especially in the cell nucleus. The nucleolus is the subnuclear organelle responsible for rRNA synthesis, processing, and assembly of the large and small ribosome subunits. Here, we have used SILAC-based quantitative proteomics to identify nucleolar SUMOylated proteins. This reveals a role for SUMOylation in the biogenesis and/or function of small nucleolar ribonucleoprotein complexes (snoRNPs) via the targeting of Nhp2 and Nop58. Using combined in vitro and in vivo approaches, both Nhp2 and Nop58 (also known as Nop5) are shown to be substrates for SUMOylation. Mutational analyses revealed the sites of modification on Nhp2 as K5, and on Nop58 as K467 and K497. Unlike Nop58 and Nhp2, the closely related Nop56 and 15.5K proteins appear not to be SUMO targets. SUMOylation is essential for high-affinity Nop58 binding to snoRNAs. This study provides direct evidence linking SUMO modification with snoRNP function.

## Highlights

► Nucleolar SUMO targets identified using SILAC-based quantitative proteomics ► K5 in Nhp2 and K467/K497 in Nop58 are SUMOylated both in vitro and in vivo ► SUMOylation may explain the distinct roles of Nop56/Nop58 and Nhp2/15.5K in snoRNPs ► SUMOylation is important for high-affinity binding of box C/D snoRNPs to snoRNAs

## Introduction

The nucleolus coordinates the machineries for transcription, processing, and maturation of ribosomal RNA (rRNA), and the assembly of ribosomal subunits. Nucleoli size and number are linked to the cellular demand for ribosome subunit production (Boisvert et al., 2007). Multiple diseases result in disruption of nucleolar integrity (Montanaro et al., 2008). Nucleolar fibrillar centers (FCs) form around tandem clusters of rRNA genes and are surrounded by the dense fibrillar component (DFC). 47S pre-rRNA production occurs at the FC/DFC border. The 47S pre-rRNA is modified and processed by multiple small nucleolar RNPs (snoRNPs; Reichow et al., 2007) to 28S, 18S, and 5.8S rRNAs mainly in the DFC. Mature rRNAs move to the granular component for assembly with 5S rRNA and ribosomal proteins. The large and small ribosome subunits are independently transported to the cytoplasm to form functional ribosomes (Boisvert et al., 2007).

Many nucleolar proteins shuttle between the nucleolus and other compartments. The steady-state “localization” of proteins to the nucleolus often results from increased retention time due to interactions with other molecules (Pederson and Tsai, 2009). The nucleolar protein database contains over 4500 proteins (NopDB; Ahmad et al., 2009), and it is clear that the nucleolus is pluripotent and possesses additional functions besides its role in ribosome subunit assembly (Boisvert et al., 2007; Pederson, 1998; Pederson and Tsai, 2009).

Small ubiquitin-like modifier (SUMO; 1–3 in humans) modification of proteins may play an important role in the nucleolus. A proportion of SUMO and related enzymes exhibit nucleolar residence (Eckert-Boulet and Lisby, 2009), including the SUMO-deconjugating sentrin-specific proteases (SENPs) 3 and 5 (Di Bacco et al., 2006; Gong and Yeh, 2006; Nishida et al., 2000). SENP3/5 and B23/NPM knockdown results in similar defects to rRNA processing (Haindl et al., 2008; Yun et al., 2008). Few nucleolar proteins have been identified as bona fide SUMO targets with an assigned function. SUMOylation can influence nucleolar localization of the target protein, such as for WRN and DNA topoisomerase-1 (Mo et al., 2002; Rallabhandi et al., 2002; Woods et al., 2004). Upregulation of the tumor suppressor CDKN2A/p14ARF may recruit SUMO2 (Haindl et al., 2008), mdm-2, and p53 (Chen and Chen, 2003; Xirodimas et al., 2002) to the nucleolus. SUMOylation of B23 (or B23-interacting proteins) antagonizes its function in ribosome biogenesis, and possibly deSUMOylation via SENP3 and/or SENP5 is needed for its function (Haindl et al., 2008; Yun et al., 2008). SUMOylation may inhibit the function of the nucleolar RNA-editing enzyme ADAR1 (Desterro et al., 2005).

SUMO1 and SUMOs 2/3 are ∼50% identical, whereas SUMO2 and -3 are ∼97% identical and often experimentally indistinguishable. SUMO proteins may have overlapping functions, given that SUMO1-deficient mice are viable (Zhang et al., 2008). However, SUMO1 and SUMO2/3 display distinct localization patterns, dynamics, preferred target proteins, propensities for chain formation, and abilities to be processed/deconjugated by SENPs (Ayaydin and Dasso, 2004; Geiss-Friedlander and Melchior, 2007; Yeh, 2009). The formation of a reversible SUMO-Lys isopeptide bond involves ATP, E1 SUMO-activating enzymes (SAE2/1), the E2 ubiquitin-conjugating enzyme 9 (Ubc9), and usually an E3 SUMO ligase. The Lys is commonly embedded in a ψ-Lys-X-Glu/Asp motif (ψ = Val, Ile, Met; X = amino acid) (Geiss-Friedlander and Melchior, 2007). SUMO modification can alter the interactions of the target, thereby affecting its stability, localization, and/or activity and influencing many different processes (Geiss-Friedlander and Melchior, 2007). Thus, SUMOylation is essential in eukaryotes and must be properly regulated for normal cellular function (Hayashi et al., 2002; Nacerddine et al., 2005; Sarge and Park-Sarge, 2009).

Here we used stable-isotope labeling by amino acids in cell culture (SILAC)-based quantitative proteomics to identify nucleolar SUMO1 and -2 targets. The major nucleolar SUMO targets were snoRNP proteins. We characterized the functional consequence of SUMOylation for Nop58 and showed it is important for snoRNP biogenesis and thus has important consequences for the production of ribosome subunits and downstream gene expression.

## Results

### A Nucleolar Pool of SUMOylated Proteins

**Figure 1:**
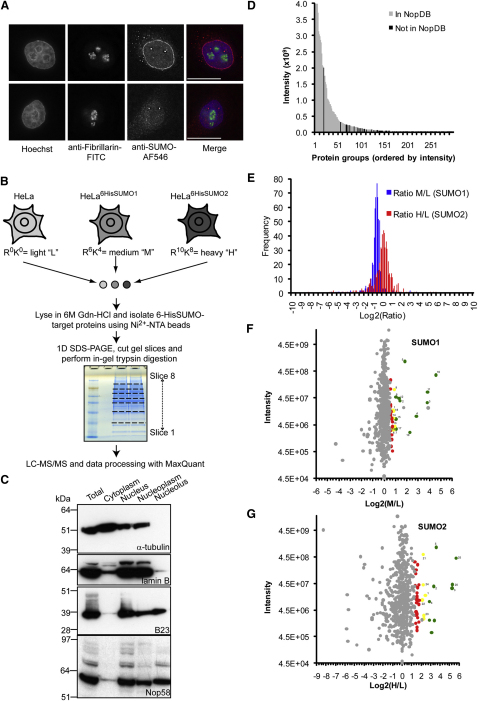
Nucleoli Contain Both SUMO1 and SUMO2/3, and Quantitative Proteomics Was Used to Identify Nucleolar SUMOylated Proteins (A) Fluorescent microscopy images of HeLa cells costained with Hoechst, anti-fibrillarin-FITC, and either anti-SUMO1-AF546 (top) or -2/3-AF546 (bottom). Separate and merged images are shown. Scale bar represents 15 μm. See also Figure S1. (B) Summary of the SILAC-based proteomics screen for nucleolar SUMO targets. HeLa and HeLa^6HisSUMO^ cells were grown in isotopically distinct media and an equal number of cells combined for fractionation (∼10^8^ total). Purified nucleoli were solubilized in 6M Gdn-HCl-containing buffer and 6HisSUMO-proteins isolated using Ni^2+^-NTA agarose before MS analysis. (C) Cell fractionation was monitored by western blotting with marker antibodies for the cytoplasm (α-tubulin), nucleus (lamin B1), and nucleolus (B23). Fractions were also probed for Nop58. Nucleolar preparations were mostly free from cytoplasmic and nucleoplasmic proteins and enriched for nucleolar proteins. (D) Column graph showing relationship between intensity and prevalence of protein groups in the current nucleolar protein database (NopDB; Ahmad et al., 2009). Only the top 300 protein groups according to intensity are shown, and the y axis is truncated to aid visualization. (E) Histograms for the frequency of log2-ratios (M/L, blue; H/L, red) obtained from the proteomic screen are distributed normally with means deviating slightly from zero due to experimental variability. (F and G) Scatter plots of total intensity of ion counts against log2 ratios (M/L and H/L, respectively). Data points are colored according to significance A (0 < green < 0.001, 0.001 < yellow < 0.01, 0.01 < red < 0.05) for protein groups with identified gene names. Reverse and contaminant protein groups are not shown. Labels (1–25) correspond to putative SUMO substrates. Unlabeled colored data points correspond to protein groups with ratio count = 1. See also Tables S1 and S2.

A proportion of SUMO1- or fluorescent protein (FP)-SUMO1-modified proteins is nucleolar (Ayaydin and Dasso, 2004; Desterro et al., 2005; Matafora et al., 2009; Figure 1A). We quantitated the levels of nucleolar SUMO1 and SUMO2/3 using immunofluorescence (IF). An ImageJ plug-in was developed for delineating nuclear and nucleolar boundaries (based on Hoechst and fibrillarin staining, respectively) and calculating the total intensity of SUMO fluorescence in each region (see Figure S1 available online). The average (nucleolar SUMO/nuclear SUMO) percent for SUMO1 and SUMO2/3 is 5.7% ± 2.1% and 5.8% ± 1.9%, respectively, based on 15 cells. The average intensity per pixel in the nucleus and the nucleolus was almost identical, suggesting that most SUMO is homogenous throughout the nucleoplasm (except for PML body foci) and nucleolus, consistent with the IF images. It was not practical to quantitate nucleolar SUMO using cell fractionation due to nonspecific SENP activity. We concluded that the high steady-state nucleolar level of SUMO justified a screen to identify specific targets.

### Screen for SUMOylated Nucleolar Proteins Using SILAC-Based Quantitative Proteomics

We used triple-encoding SILAC (Figure 1B) for analysis of the nucleolar SUMO proteome (Ong et al., 2002). HeLa cell lines that stably express 6HisSUMO enabled purification with Ni^2+^-NTA beads under denaturing conditions to minimize SENP activity (Tatham et al., 2009). Nucleoli were isolated from the combined cells before target purification and were highly purified as confirmed by western blots (Figure 1C) and light microscopy (data not shown). MaxQuant analysis quantitated 573 out of the 590 identified protein groups (excluding hits in the reverse database; Table S1). Comparison of identified proteins in either input (data not shown) or eluate (Figure 1D) samples with the >4500 proteins identified in purified nucleoli (Ahmad et al., 2009) confirmed that most protein groups with high intensity appear in the NopDB (http://www.lamondlab.com/NOPdb3.0/).

Histograms (M/L and H/L, Figure 1E) reveal that log2-ratios are normally distributed due to the lower levels of nucleolar 6HisSUMO-tagged proteins (log2-ratio > 0) and environmental contaminants (log2-ratio < 0), compared to proteins that bind nonspecifically to the Ni^2+^-NTA resin (log2-ratio∼0). Thus, we could use the MaxQuant-derived significance scores to identify targets (the probability of obtaining a particular ratio given that the distribution of log-ratios has normal upper and lower tails [Cox and Mann, 2008]), where a low score corresponds to a likely SUMO substrate. Scatter plots (total intensity versus log2-ratio) for each protein group are shown (M/L in Figure 1F; H/L in 1G), with data points colored according to significance A. We used filters of significance A <0.01 and ratio count >1 to identify 19 SUMO1 and 11 SUMO2 putative substrates, with 5 proteins common to both lists (1–25; Figures 1F and 1G; Tables 1 and 2).

Tryptic peptides from input samples (mixed L/M/H whole-cell lysates prior to fractionation) were also analyzed by LC-MS/MS (Table S2). Since protein levels should be similar in all three cell lines before SUMO target enrichment, low significance scores indicate proteins that are sensitive to overexpression of 6HisSUMO and are thus present at different levels in HeLa^6HisSUMO^ cells (Tables 1 and 2).

Four candidates for nucleolar SUMOylation, Nhp2, Nop58, DKC1, and NOLC1 (with sequence coverage 39.9%, 53.7%, 24.5%, and 8.2%, respectively; Tables 1 and 2), are either members of snoRNP complexes or involved in their biogenesis (Filipowicz and Pogacic, 2002; Reichow et al., 2007). We characterised the two proteins with highest sequence coverage, i.e., Nhp2 and Nop58, to examine whether SUMOylation plays a role in snoRNP formation and/or function.

### Nhp2 and Nop58 Are Substrates for SUMOylation

**Figure 2:**
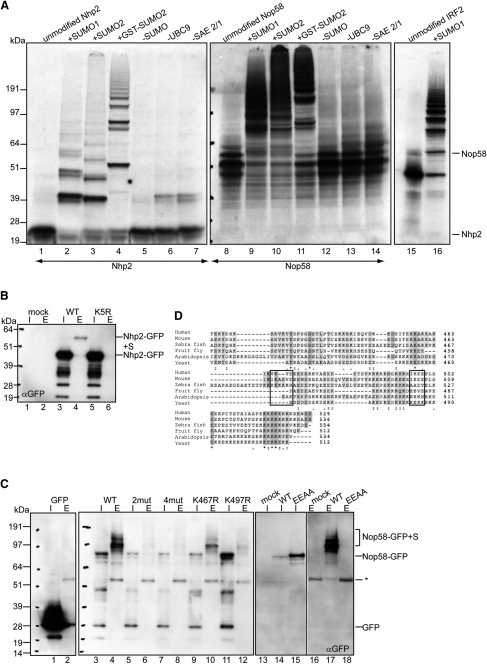
Nhp2 and Nop58 Are SUMO Substrates, and Identification of Modification Sites (A) ^35^S-Met-labeled Nhp2, Nop58, and IRF2 (positive control) were subjected to in vitro SUMO assays. Reaction conditions were identical for lanes 2, 9, and 16; 3 and 10; and 4 and 11. The positions of unmodified Nhp2 and Nop58 are indicated. (B) HeLa^6HisSUMO2^ cells were transfected (48 hr) with either WTNhp2- or K5R-Nhp2-GFP and lysed in 6M Gdn-HCl. 6HisSUMO2 conjugates were purified on Ni^2+^-NTA agarose. Input (I) and eluate (E) samples were analyzed by anti-GFP western blotting. The positions of Nhp2-GFP and Nhp2-GFP+SUMO are indicated. Blot was reprobed with anti-SUMO2 (Figure S2A). (C) As in (B), except with GFP, WTNop58-, 2mutNop58 (K467R, K497R)-, 4mutNop58 (K390R, K415R, K467R, K497R)-, K467RNop58-, K497RNop58-, or EEAANop58 (E469A, E499A)-GFP. The positions of background bands (^∗^), Nop58-GFP, and Nop58-GFP+SUMO are indicated. Lanes 1–12 were reprobed with anti-SUMO2 (Figure S2B). (D) Nop58 C-terminal sequences from different species (human, Q9Y2X3; mouse, Q6DFW4; zebrafish, Q6P6X6; fruit fly, Q9VM69; *Arabidopsis*, O04658; and yeast, Q12499) were aligned using the ClustalW program (Uniprot). Human Nop58 SUMO sites are boxed.

To verify that Nhp2 and Nop58 are SUMO substrates, ^35^S-Met-labeled Nhp2 and Nop58 were subjected to in vitro SUMO modification assays (Desterro et al., 1997; Tatham et al., 2001). Figure 2A shows that unmodified Nhp2 migrates at ∼17 kDa, whereas Nop58 migrates as a series of bands at ∼60 kDa, likely due to internal translational initiation (Nelson et al., 2000). The majority of both Nhp2 and Nop58 migrated more slowly after in vitro SUMOylation (Figure 2A; lanes 2–4 and 9–11), dependent upon addition of SUMO, Ubc9, and SAE2/1 (lanes 5–7 and 12–14). Preliminary work suggests the double bands for Nhp2 are not due to phosphorylation (data not shown). We conclude that both Nhp2 and Nop58 contain motifs that are suitable for SUMOylation.

We next tested whether Nhp2 and Nop58 are substrates for SUMOylation in vivo. Transfections of HeLa^6HisSUMO2^ cells with plasmids encoding either Nhp2-GFP or Nop58-GFP, followed by denaturing Ni^2+^-NTA pull-downs, revealed higher MW forms of both Nhp2- and Nop58-GFP in the eluates compared to input samples (Figures 2B and 2C; lanes 3 versus 4), consistent in size with SUMOylation. Control pull-downs were performed with either mock transfections or empty plasmid (Figures 2B and 2C; lanes 1 and 2). We conclude that Nhp2 and Nop58 can be SUMOylated both in vivo and in vitro.

### Identification of SUMO Modification Sites in Nhp2 and Nop58

**Figure 3:**
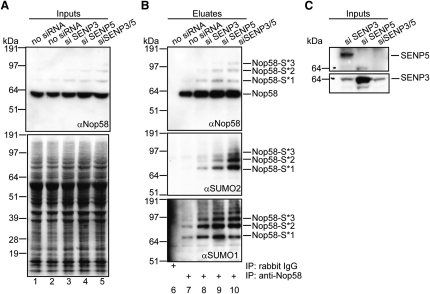
Detection of Endogenous SUMOylated Nop58 in Absence of Exogenous SUMO and deSUMOylation of Nop58 by the Nucleolar SENPs U2OS cells were transfected (72 hr) with siRNAs against SENP3 and/or SENP5 and lysed initially in 1% SDS. Endogenous Nop58 was isolated using control or anti-Nop58 antibodies (B). Inputs (A and C) and eluted proteins (B) were analyzed by western blotting using anti-Nop58 (A and B, top), -SUMO2 (B, middle), -SUMO1 (B, bottom), -SENP5 (C, top), or -SENP3 (C; bottom). Ponceau staining was used as a loading control (A, bottom). The positions of bands corresponding to Nop58 and Nop58-SUMO are indicated. See also Figure S3.

Putative SUMO sites were identified by inspection of Nhp2 (Q9NX24) and Nop58 (Q9Y2X3) sequences using SUMOplot (Abgent), namely K5^∗^ for Nhp2 and K390, K415, K467^∗^, and K497^∗^ for Nop58 (asterisk indicates the closest match to consensus). Single, double (2mutNop58 = K467R and K497R) or quadruple (4mutNop58) mutations were made in the GFP-fused proteins, expressed in HeLa^6HisSUMO2^ cells, and purified by denaturing Ni^2+^-NTA pull-downs (Figures S2A and S2B). The lack of K5R-Nhp2 SUMOylation indicated that K5 is its major SUMO site (Figure 2B; lane 6). The K467R and K497R double mutation in Nop58-GFP (Figure 2C; lane 6) abrogated its SUMOylation. Inspection of the single mutations (Figure 2C; lanes 10 and 12) showed that K497 is more important than K467 for SUMOylation of Nop58-GFP; however, both mutations are required for near-complete loss of this modification. These results were consistent with in vitro SUMOylation assays for Nop58 mutants (Figure S2C). Equivalent experiments using HeLa^6HisSUMO1^ cells did not enrich for SUMOylated Nhp2- or Nop58-GFP (data not shown). However, we detected endogenous Nop58 modified by SUMO1 after Ni^2+^-NTA pull-downs using cytoplasmic, nucleoplasmic, and nucleolar extracts from these cells (Figure S2D; also Figure 3 for Nop58-SUMO1). This revealed that the majority of SUMOylated Nop58 resides in the nucleolus rather than the nucleoplasm.

To confirm that K467 and K497 in Nop58 are directly conjugated to SUMO and do not mediate distal modifications (Tatham et al., 2009), a double Glu to Ala mutant was generated (EEAANop58-GFP = E469A and E499A) and analyzed as above (Figure 2C; lanes 13–18). The almost complete loss of SUMOylation for EEAANop58-GFP provides rigorous evidence that K467 and K497 in Nop58 are directly conjugated to SUMO2. Alignment of primary Nop58 sequences from different species reveals that K467, K497, and the surrounding consensus motif for SUMOylation (ψ-K-X-[E/D]) are conserved between human and mouse, but not in lower organisms, suggesting that the ability of Nop58 to be SUMOylated has appeared recently in evolution (Figure 2D).

### Detection of Endogenous Nop58 SUMOylation

To test whether endogenous Nop58 could be SUMOylated in the absence of exogenous SUMO, we immunoprecipitated Nop58. SENP activity was minimised by lysing cells in 1% SDS with 10 mM IAA before adding DTT. Lysates made after 72 hr knockdown of SENP3 and/or -5 were also used (Figure 3C). The inputs and eluates from the IPs (Figures 3A and 3B) were analyzed by western blotting, and Ponceau staining was used as a loading control (Figure 3A; bottom). Unmodified Nop58 was enriched in the eluates from the anti-Nop58 IPs (Figure 3B; top), along with slower migrating bands enriched after knockdown of SENP3 and/or SENP5. Since these bands react with antibodies against SUMO, we conclude that they correspond to Nop58 with one, two, or three SUMOs attached (Figure 3B; middle and bottom). Band intensities in the anti-SUMO blots should not be compared directly due to different affinities of the primary antibodies. Quantitative Western blotting using mixed anti-SUMO1 and -2/3 antibodies to measure the total signal ratios of lane 6 versus 1 (background) and 7 versus 2 suggested that the percentage of total SUMOylation contributed by Nop58 was >0.00002% (data not shown). Finally, Nop58 SUMOylation did not change in response to different cellular stress conditions (Figure S3). In summary, Nop58 is a physiological SUMO substrate, even in the absence of exogenous SUMO, and nucleolar SENPs may be involved in modulating Nop58 modification levels.

### Nop56 and 15.5K Are Poor SUMO Substrates Compared to Nop58 and Nhp2

Nop58 associates with Nop56, fibrillarin, and 15.5K/nhpx within box C/D snoRNPs, and Nhp2 with DKC1, Nop10, and Gar1 within box H/ACA snoRNPs (Reichow et al., 2007). Despite ∼40% sequence identity between Nop58 and Nop56, and Nhp2 and 15.5K, we only identified Nop58 and Nhp2 as nucleolar SUMO targets. To determine if homologous snoRNP components have acquired different propensities for SUMOylation, we investigated if Nop56 and 15.5K are SUMOylated.

**Figure 4:**
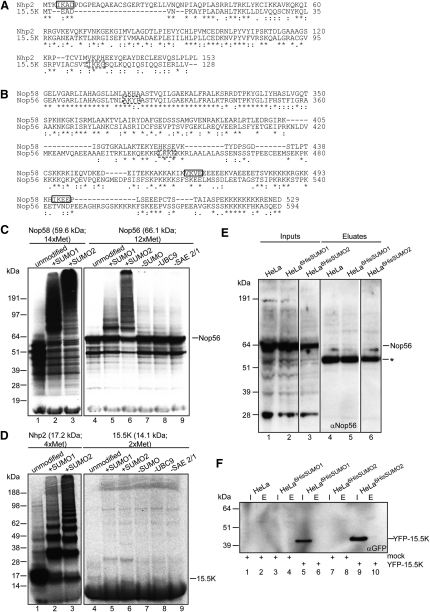
Nop56 and 15.5K Are Poor SUMO Substrates (A) Nhp2 (Q9NX24) and 15.5K (P55769) full-length sequences were aligned using the ClustalW program (Uniprot). Validated SUMO site in Nhp2 is boxed. (B) As in (A), but with Nop56 (O00567; aa 301–594) and Nop58 (Q9Y2X3; aa 291–529). The predicted SUMO sites (A and B; SUMOplot) are shown (dashed boxes). (C and D) ^35^S-Met-labeled Nop58, Nop56, Nhp2, and 15.5K were subjected to identical in vitro SUMO assays. The numbers of Met residues, MWs, and positions of unmodified Nop56 and 15.5K are indicated. (E) Lysates from HeLa, HeLa^6HisSUMO1^ and HeLa^6HisSUMO2^ cells (inputs; lanes 1–3) were subjected to Ni^2+^-NTA pull-downs (eluates; lanes 4–6), and analyzed by western blotting with anti-Nop56 and anti-Nop58 (Figure S4A). Asterisk corresponds to background bands. (F) HeLa, HeLa^6HisSUMO1^, and HeLa^6HisSUMO2^ cells were transfected with YFP-15.5K as indicated and subjected to Ni^2+^-NTA pull-downs. Input (I) and eluate (E) samples were analyzed by western blotting with anti-GFP and anti-SUMO (Figure S4B).

The SUMOylation sites in Nop58 (K467 and K497) and Nhp2 (K5) are not well conserved in Nop56 or 15.5K, respectively (Figures 4A and 4B). The abilities of Nop58 and Nop56, and Nhp2 and 15.5K, to act as in vitro SUMO substrates were compared (Figures 4C and 4D; lanes 2 and 3 versus 5 and 6). Although some SUMOylation for Nop56 and 15.5K can be seen, these modifications are less efficient than for Nop58 or Nhp2, respectively. Given that SUMOylation of nonphysiological targets can occur in vitro, we also investigated the abilities of endogenous Nop56 (Figure 4E) or transiently expressed YFP-15.5K (Figure 4F) to be modified by HisSUMO in vivo using denaturing Ni^2+^-NTA pull-downs. Unmodified Nop56 and YFP-15.5K were identified in the input samples but SUMOylated forms were not enriched in the eluate samples. These blots were reprobed with either anti-Nop58, -SUMO1 or -SUMO2 as positive controls for the pull-downs (Figure S4). Together, these results demonstrate that Nop56 and 15.5K are poor SUMO substrates compared to Nop58 and Nhp2.

### SUMOylation Is Not Essential for Subnuclear Localization of Nop58 or Interaction with Fibrillarin

**Figure 5:**
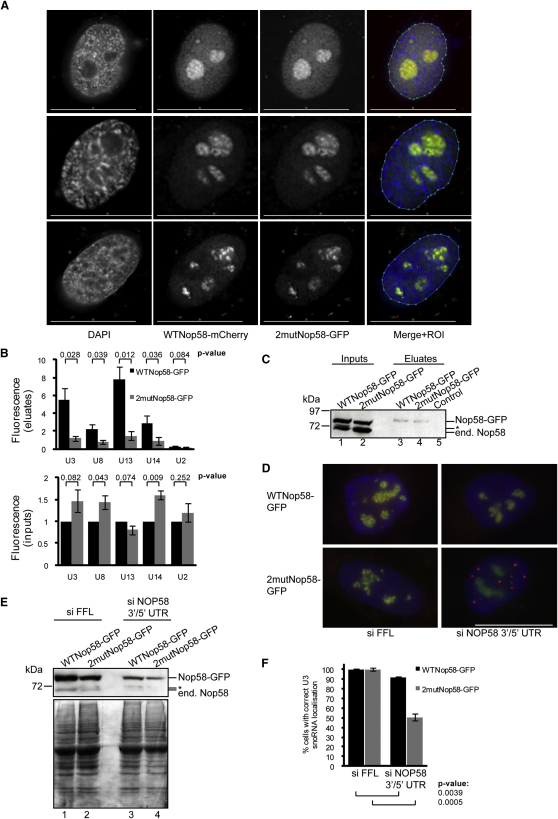
Toward Identification of the Functional Outcome of Nop58 SUMOylation (A) Twenty-four hour cotransfection of U2OS cells with constructs encoding WTNop58-mCherry and 2mutNop58-GFP and fixed-cell fluorescence microscopy reveals that SUMOylation does not alter the subcellular localization of Nop58, consistent with proper incorporation into snoRNPs. Each horizontal row corresponds to a single z plane. Separate and merged images (with ROI used for quantitation) are shown. Scale bar represents 30 μm. See also Figures S5A–S5C. (B and C) Nop58 SUMOylation is important for snoRNA binding, as shown by qPCR detection of snoRNAs and U2 snRNA (control) in eluate (top) and input (bottom) samples from anti-GFP IPs using U2OS^WTNop58-GFP^ and U2OS^2mutNop58-GFP^ lysates. Each column/error bar represents the average/standard deviation of measurements from three different IPs. GAPDH mRNA was used as a reference for normalization. Input measurements were adjusted so values for WTNop58-GFP = 1. Eluate measurements represent fold enrichment compared to control IPs (Protein-G/anti-HA Sepharose beads). P values were obtained from two-tailed, heteroscedastic t tests. Input and eluate samples were analyzed by western blotting (C) with anti-Nop58 for endogenous and FP-tagged Nop58. Asterisk corresponds to background bands. See also Figures S5D–S5F. (D–F) U2OS^WTNop58-GFP^ and U2OS^2mutNop58-GFP^ cells were cotransfected (40 hr) with a rat U3B.7 snoRNA-encoding plasmid and either control (FFL) or endogenous Nop58 siRNAs targeted to 5′ and 3′ untranslated regions (UTRs). Knockdown was confirmed by western blotting (E) with anti-Nop58 (top) and Ponceau staining as a loading control (bottom). Asterisk corresponds to background bands. Cells were hybridized in situ with a Cy5-conjugated oligonucleotide probe (red) against rat U3B.7 snoRNA and stained with DAPI (blue). Merged images corresponding to a single z plane are shown (D). Scale bar represents 30 μm. The average percent of cells (based on 40 cells in three different experiments) with correct rat U3B.7 snoRNA localization was quantitated before and after Nop58 knockdown (F), where siFFL values have been adjusted to be 100%, and the P values and error bars obtained as in (B).

After nuclear import, the core box C/D snoRNP proteins localize to the nucleoplasm and Cajal bodies (CBs) to assemble with box C/D snoRNAs before the mature snoRNP is transported to the nucleolus (Filipowicz and Pogacic, 2002). Indeed, endogenous Nop58 was found to be enriched in nucleoplasmic and nucleolar fractions (Figure 1C). To compare the localization of WTNop58-GFP with the non-SUMOylatable 2mutNop58-GFP, equal amounts of plasmids encoding either WTNop58-mCherry or 2mutNop58-GFP were cotransfected (confirmed by western blotting; data not shown) and cells examined by fixed-cell fluorescence microscopy. Images from three cells with varying amounts of Nop58-FP (Figure 5A) demonstrate that WTNop58-GFP and 2mutNop58-GFP are present in nucleoli, CBs, and the nucleoplasm (Figure S5D shows fibrillarin and coilin staining). The amount of colocalization was quantitated by calculating the Pearson coefficient of colocalization for cell nuclei (the ROI; Zinchuk et al., 2007), and values of 0.9619, 0.9685, and 0.9475 confirm an almost identical localization of WT- and 2mutNop58-GFP. Similar results were obtained by cotransfection of HaCaT cells (data not shown). The redistribution of WT- and 2mutNop58-GFP to nucleolar caps (Lechertier et al., 2009) after transcriptional inhibition was also identical (Figure S5A). We conclude that SUMOylation does not prevent the steady-state subnuclear localization of Nop58.

The localization of 2mutNop58-FP suggested that it is incorporated into snoRNPs, and IPs of endogenous fibrillarin from cells transiently cotransfected with WTNop58-mCherry and/or 2mutNop58-GFP showed that the Nop58-FPs were isolated at similar levels (Figure S5B, lane 8). We also examined the amount of fibrillarin coimmunoprecipitated from U2OS cell nuclei stably expressing either WTNop58- or 2mutNop58-GFP (below) after anti-GFP IPs (Figure S5C). Quantitative western blotting revealed ∼40% less fibrillarin associated with 2mutNop58-GFP compared to WTNop58-GFP. The different results regarding fibrillarin association are likely due to Nop58-FP expression levels and the time required for newly expressed proteins to be incorporated into snoRNPs. Taken together, we conclude that SUMOylation is important, but not essential, for incorporation of Nop58 into snoRNPs.

### SUMOylation Modulates the Interaction of Nop58 with snoRNAs and Is Essential for Their Nucleolar Localization

We investigated the effect of SUMO on association of Nop58-GFP with snoRNAs. U2OS cell lines were generated that stably express either WT- or 2mutNop58-GFP (U2OS^WTNop58-GFP^ or U2OS^2mutNop58-GFP^, respectively). Fluorescence microscopy using markers for the DFC (fibrillarin) and CBs (coilin) confirmed that the stably expressed FPs localized correctly (Figure S5D; data not shown). Both FPs migrated at the expected MW (90 kDa; Figure S5E), and flow cytometry showed that >90% of both cell lines was GFP positive (data not shown). Measurement of cellular DNA content showed that exogenous expression of WT- or 2mutNop58-GFP had minimal effects on cell-cycle progression (Figure S5F).

qPCR to detect RNA levels in U2OS^WTNop58-GFP^ and U2OS^2mutNop58-GFP^ eluates (Figure 5B; top; values adjusted so control IP = 1) revealed that all four box C/D snoRNAs tested, but not the control U2 snRNA, were enriched after anti-GFP IPs. Strikingly, this enrichment was significantly reduced (p values <0.05) for the 2mut- compared to WTNop58-GFP IPs. This reduction was not due to a change in snoRNA levels, since U2OS^2mutNop58-GFP^ inputs (Figure 5B; bottom) contained either equal (U3, U13) or higher levels (U8, U14) compared to U2OS^WTNop58-GFP^ inputs. The reason for higher U8 and U14 snoRNA levels in U2OS^2mutNop58-GFP^ cells is not clear. U17, U19, and U64 box H/ACA snoRNA levels were also unchanged (data not shown). Input and eluate samples were analyzed by western blotting to confirm that WTNop58- and 2mutNop58-GFP were isolated with similar efficiencies (Figure 5C; lanes 3 versus 4). Exogenous SENP3/5 expression did not alter Nop58-GFP snoRNA binding (data not shown), which suggests that SENP3/5 activity is not rate limiting in determining the relative levels of SUMOylated and non-SUMOylated Nop58 and is consistent with the observed robust activity of SENP3/5 toward Nop58 (Figure 3). Together, these results suggest that box C/D snoRNP complexes that contain 2mutNop58-GFP have a lower affinity for snoRNAs.

To investigate if Nop58 SUMOylation is required for the localization of box C/D snoRNAs to the nucleolus, we examined the localization of transiently expressed rat U3B.7 snoRNA (Verheggen et al., 2002), which could be detected specifically via fluorescence in situ hybridization (FISH) (Figure 5D). The average percent of cells (based on 40 cells in three different experiments) with correct rat U3B.7 localization after Nop58 depletion was quantitated for U2OS^WTNop58-GFP^ and U2OS^2mutNop58-GFP^ cells. Specific endogenous Nop58 knockdown was achieved using siRNAs targeted to noncoding regions absent in the Nop58-GFP plasmids (Figure 5E). We observe that loss of endogenous Nop58 can result in decreased Nop58-FP levels, for unclear reasons. Importantly, replacement of endogenous Nop58 with 2mutNop58-GFP caused a more statistically significant decrease (49.6% ± 5.3%) in cells with correct rat U3B.7 localization compared to an 8.3% ± 1.6% decrease using WTNop58-GFP (Figure 5F). The majority of rat U3B.7 snoRNA localized to nucleoplasmic foci that likely correspond to transcription sites (Verheggen et al., 2002). WTNop58- and 2mutNop58-GFP remained localized to CBs and nucleoli after endogenous Nop58 knockdown. We conclude from the above IF and IP/qPCR experiments that SUMOylation of Nop58 is essential for high-affinity snoRNA binding and the localization/retention of box C/D snoRNAs, such as U3, in the nucleolus.

## Discussion

We report an unbiased, high-throughput characterization of nucleolar SUMO targets. Rather than using a candidate target approach, SILAC-MS enables the relative importance of SUMOylation for all nucleolar processes to be ascertained simultaneously. SILAC-MS data identified multiple nucleolar SUMOylated proteins (Tables 1 and 2). Many of these targets contain predicted SUMOylation sites and copurify with nucleoli (Ahmad et al., 2009; Figure 1D). Some have been identified in other SUMO proteomic screens or experimentally verified as a SUMO target (i.e., #12 = FHL1; Wang et al., 2007). Quantitative IF analysis indicated that ∼6% of the total nuclear pools of both SUMO1 and SUMO2/3 is nucleolar. MS analysis was also performed on whole-cell extracts prior to fractionation for the mixed HeLa and HeLa^6HisSUMO^ cells to control against proteins present at higher levels as a result of SUMO overexpression. Consequently, several putative targets were not pursued because they appeared at higher levels in the HeLa^6HisSUMO^ cells, including CDKN2A/p14ARF (#20), which has a low H/L significance A score (1.20E-11) but also a low score for its input whole H/L ratio (5.67E-18). CDKN2A promotes the SUMOylation of many proteins, such as p53 (Chen and Chen, 2003), mdm-2 (Xirodimas et al., 2002), and WRN (Woods et al., 2004). Regulation of CDKN2A stability has been linked to ubiquitin (Chen et al., 2010) but not SUMO, suggesting an undiscovered mechanism exists for either direct or indirect SUMO-mediated stabilization of CDKN2A.

Two of the most promising nucleolar SUMO targets were Nhp2 and Nop58, i.e., proteins with the highest M/L and H/L ratios, sequence coverage (>40%), and no significant enrichment in the input samples. These are core members of box H/ACA and box C/D snoRNPs, respectively (Reichow et al., 2007).

Both in vitro SUMOylation assays and in vivo cotransfection experiments show that Nop58 and Nhp2 are substrates for SUMOylation. Mutational analyses identified the SUMO-modified residues as K5 in Nhp2, and K467 and K497 in Nop58. Nop58 was shown to be a physiological SUMO substrate because SUMOylation of endogenous Nop58 was detected in the absence of exogenous SUMO (Figure 3). Endogenous SUMOylation is often difficult to demonstrate due to the substoichiometric level of this modification, which can still impart major biological consequences (Geiss-Friedlander and Melchior, 2007). The steady-state level of Nop58 SUMOylation in vivo is determined by the opposing activities of the SUMO-conjugation machinery and SENPs. Nop58 is likely a target for the nucleolar SENP3 and/or -5, since SENP3/5 knockdown increased SUMOylated Nop58 levels. This may be needed for snoRNP function in rRNA processing, analogous to reports for SENP3/5 and B23 (Haindl et al., 2008; Yun et al., 2008). Nop58 SUMOylation occurs during normal cell growth conditions and not only after stress. In contrast to most SUMOylated proteins (Geoffroy and Hay, 2009; Golebiowski et al., 2009), Nop58 SUMOylation did not increase after either heat shock or MG132 treatment. These data suggest cycles of SUMO conjugation and deconjugation are linked with physiological processes affecting snoRNP assembly and/or function.

We propose that Nop58 is predominantly conjugated with SUMO2/3, rather than SUMO1, although this will need to be formally tested. SENP3/5 display specific isopeptidase activity toward SUMO2/3 conjugates (Yeh, 2009), and SUMOylated Nop58 increases after SENP3/5 knockdown. Further, Nop58 attached to three SUMOs was detected but only two Nop58 SUMO sites were identified, suggesting that a SUMO chain—a specific property of SUMO 2/3—forms at least on one Lys residue. Taken together, this suggests that Nop58 is preferentially modified by SUMO2/3.

Although the majority of SUMOylated Nop58 is detected in the nucleolus, future work will determine whether SUMO conjugation and/or deconjugation also occur here. For example, the enzymatic attachment of SUMO to Nop58 could occur in the nucleoplasm with the modified form accumulating in the nucleolus. Nop58 is assembled into snoRNPs in the nucleoplasm and CBs (Filipowicz and Pogacic, 2002), and snoRNP proteins can shuttle continually between the nucleoplasm and nucleolus (Leary et al., 2004). It will be interesting to map the SUMOylation/deSUMOylation cycle onto the pathway of snoRNP biogenesis. Consistent with a role for SUMOylation in this pathway, we show that SUMO modification of Nop58 affects, either directly or indirectly, high-affinity binding to box C/D snoRNAs and the localization of newly synthesized snoRNA to the nucleolus (Figure 5). Under conditions where endogenous Nop58 is mostly replaced by 2mutNop58, newly synthesized U3 snoRNA form nucleoplasmic foci. Given that 2mutNop58-GFP remains nucleolar, it is possible that assembly factors are not efficiently recycled in these cells. Alternatively, defects in 2mutNop58-GFP localization may be observed after a longer time. The ability of snoRNP proteins to localize correctly to the nucleolus despite a reduced affinity for snoRNAs has been reported previously for fibrillarin (Romanova et al., 2009).

The level of Nop58 (both unmodified and modified) may increase after SENP3/5 knockdown (Figure 3B), suggesting that SUMOylation affects its stability. Links between SUMOylation, ubiquitin, and proteasomal degradation have been reported (Geoffroy and Hay, 2009). Our preliminary data investigating compartment-specific turnover rates of proteins (F.-M. Boisvert and A.I.L., data not shown) show a longer half-life of nucleolar compared to nucleoplasmic Nop58 (∼37 versus 15 hr), indicating that the SUMOylation/deSUMOylation cycle for Nop58 increases its stability. This is consistent with a report that truncated (and thus possibly non-SUMOylated) Nop58 in yeast is less stable than the full-length version (Wu et al., 1998). Loss of SUMOylation has some impact on overall stability of the box C/D snoRNP complex, with ∼1.7-fold less fibrillarin associated with 2mutNop58- compared to WTNop58-GFP. However, comparison with the decrease in its association with snoRNAs (∼4.1 fold) suggests that the major role of Nop58 SUMOylation is to promote high-affinity snoRNA binding.

It seems that specific components of box C/D and H/ACA snoRNPs have evolved as preferential SUMO targets. Nop58 is highly related to Nop56, and a single Nop56/58-like protein exists in Archaea (Reichow et al., 2007). However in eukaryotes, Nop56 and Nop58 display separable functions in the box C/D snoRNP, e.g., Nop58 but not Nop56 is required for snoRNA stability in vivo (Reichow et al., 2007), neither protein can complement the other, and each is involved in the synthesis of different ribosomal subunits (Gautier et al., 1997; Wu et al., 1998). Similarly, Nhp2 and 15.5K/nhpx are homologous and are related to the archael L7Ae protein, yet exhibit different RNA-binding characteristics and incorporation into RNPs (e.g., box C/D snoRNP and U4 snRNP for 15.5K, box H/ACA snoRNP and telomerase for Nhp2; Reichow et al., 2007; Schultz et al., 2006; Wang and Meier, 2004). We show that Nop58 and Nhp2 have evolved as preferential SUMO targets, suggesting additional differences in the assembly pathways for box C/D and H/ACA snoRNPs. Our quantitative proteomics screen failed to identify Nop56 and 15.5K as substrates for SUMOylation, and neither was detected linked to 6HisSUMO in vivo using Ni^2+^-NTA pull-downs. In vitro SUMO assays detected some level of SUMO attachment, consistent with a recent report that Nop56 is SUMOylated (Matafora et al., 2009), but confirmed that both proteins are poor SUMO substrates relative to Nop58 and Nhp2.

Differences in posttranslational modifications (PTMs) such as SUMOylation of K467 and K497 could underlie the unique roles of Nop56 and Nop58, and the highly charged Nop58 C-terminal “tail” (∼aa 441–529) could act as a sensor to regulate the function and/or level of Nop58 via PTMs. Indeed, Matic et al. (2010; in this issue of *Molecular Cell*) have reported that casein kinase II-mediated phosphorylation of Nop58 S502 is critical for its SUMOylation. Nop58 (aa 482–529) and Nop56 (aa 505–603) tails exhibit nucleolar and CB localization, and truncated Nop58 (aa 1–482) and Nop56 (aa 1–505) reside in the cytoplasm (C.V., unpublished data). This suggests the Nop58 and Nop56 tails each contain a NoLS, and are involved in regulating the biogenesis and/or transport of box C/D snoRNPs to the nucleolus. The Nop58 tail is rich in Lys (29/89) and Glu (22/89) residues and predicted to be basic (theoretical pI = 9.4). Molecular interactions of the tail may need to be regulated by modulating the accessibility of these charged residues, e.g., by the addition of acidic SUMO and/or phosphate moieties. A consequence of the loss of this modulation is abrogation of high-affinity binding to box C/D snoRNAs, since we observe a reduction in the amounts snoRNA that copurify with similar levels of WT and non-SUMOylatable Nop58. This was true for both intron-encoded (U14) and RNA polymerase II-transcribed snoRNAs (U3, U8, U13; Filipowicz and Pogacic, 2002). Finally, we show that following replacement of endogenous Nop58 with a non-SUMOylatable version, newly synthesized U3 snoRNAs do not localize correctly to nucleoli. This suggests that the reduced affinity of non-SUMOylatable Nop58 for snoRNAs abrogates box C/D snoRNP biogenesis.

In summary, we have identified a role for SUMO2/3 modification in the mechanism of box C/D snoRNP biogenesis, acting via Nop58. A proteomic screen identified multiple nucleolar SUMO targets, and it will be important to characterize which of these modifications may also have mechanistic significance. For example, Nhp2 is a core box H/ACA snoRNP protein that we showed to be SUMOylated on K5 by in vivo and in vitro assays. It will be interesting in the future to examine whether SUMOylation can also regulate the assembly and/or function of box H/ACA snoRNPs and possibly other nuclear RNP complexes.

## Experimental Procedures

### Plasmid Constructs and Mutagenesis

Constructs were generated using standard techniques (Supplemental Information). The 6HisSUMO and rat U3B.7 plasmids are described in Tatham et al. (2009) and Verheggen et al. (2002). pcDNA3-IRF2 was a gift (R. Hay). Sequences of oligos/plasmids available on request.

### Cell Culture, Transfections, and Drug Treatments

Cell lines were cultured using standard protocols using selective markers (200 μg/ml G418 or 1 μg/ml puromycin) as appropriate. Effectene, Polyfect (QIAGEN), Lipofectamine RNAiMAX or −2000 (Invitrogen) was used for plasmid/siRNA transfections (Supplemental Information).

### Immunofluorescence

Cells were fixed in either 3.7% PFA/PHEM buffer (Figure 1A, Figure S1) or 3.7% PFA/PBS for 7–10 min (Figure 5A, Figure S5), permeabilised with 1% Triton X-100/PBS (10–15 min), blocked with either 0.2% fish gelatine/PBS (Figure 1A, Figure S1) or 1% goat serum/PBS (Figure 5A, Figure S5) for 10–30 min, incubated with antibodies (1 hr), washed, stained (25 μg/ml Hoechst or 1 μg/ml DAPI), and mounted onto slides in VectorShield (Vector Lab). Primary antibodies were mouse anti-fibrillarin (Abcam; 1:500), sheep anti-SUMO1 (1:300), and anti-SUMO2 (1:100; both gifts from R. Hay). Secondary antibodies were all from Jackson Immunochemicals or Molecular Probes (Alexa Fluor 546-conjugated donkey anti-sheep). Images were acquired with a DeltaVision Spectris wide field deconvolution microscope/Olympus IX71 stand/60× 1.42 NA lens. Cells were exposed to provide an intensity of ∼2000 counts (12 bit). Processing and analysis was done using SoftWorx (Applied Precision).

### Fluorescence In Situ Hybridization

Cells were fixed (4% PFA/PBS; 20 min) and incubated in 70% ethanol (24 hr; 4°C). FISH was done according to Samarsky et al. (1998) with a Cy3-labeled oligonucleotide against rat U3B.7 snoRNA (Verheggen et al., 2002) and mounted in 90% glycerol/PBS containing PPD (1 mg/ml) and DAPI at pH 8.0. Images were captured on a Leica DMRA microscope equipped for epifluorescence using a 100× 1.3 NA lens and analyzed with the Metamorph acquisition software.

### Quantitation of Nucleolar SUMO Immunofluorescence

The microscope was calibrated for uniformity of fluorescence field, chromatic shift, and spectral bleedthrough, and no detectable cross-reactivity was observed between the antibodies used. Fifty 0.2 μm-spaced images per cell were acquired in the z plane. An in-house ImageJ plug-in (P. Schofield; available on request) identified nuclear and nucleolar borders for a single cell in each deconvolved, z stack image using connection threshold segmentation with manual threshold adjustment (Supplemental Information).

### SILAC Labeling

HeLa and HeLa^6HisSUMO^ cells were grown for at least five to six cell doublings in media containing labeled amino acids (Cambridge Isotope Lab) as follows: R^0^K^0^ (L-arginine and L-lysine), R^6^K^4^ (L-arginine ^13^C and L-lysine 4,4,5,5-D4) or R^10^K^8^ (L-arginine ^13^C/^15^N and L-lysine ^13^C/^15^N) (Trinkle-Mulcahy et al., 2008).

### Preparation of SILAC-Nucleolar Extracts, Purification of 6HisSUMO-Conjugated Proteins, LC-MS/MS, and Data Analysis

Equal numbers of labeled cells were mixed and nucleoli were isolated (Andersen et al., 2002) in the presence of 10 mM IAA. Purified nucleoli were solubilised in 6M Gdn-HCl-containing lysis buffer.

6HisSUMO-conjugated proteins were purified using Ni^2+^-NTA agarose (QIAGEN) (Tatham et al., 2009). Proteins were reduced and alkylated prior to SDS-PAGE. Lyophilized peptides in 1% formic acid were analyzed by LC-MS/MS using an LTQ-Orbitrap mass spectrometer (Thermo Fisher Scientific; Trinkle-Mulcahy et al., 2008). Data were analyzed with MaxQuant (v1.0.13.13; Cox and Mann, 2008), Mascot (Matrix Science, v2.2.2), and the human IPI database (v3.52; Kersey et al., 2004). Default parameters were used in the Identify and Quant modules except that the minimum ratio count was set to 1 for protein quantification.

### In Vitro SUMO Modification Assays

Recombinant SAE2/1, Ubc9, SUMO1, SUMO2, GST-SUMO2 (all gifts, R. Hay; Desterro et al., 1997; Tatham et al., 2001), radiolabeled substrates (TNT Quick Coupled Transcription/Translation System; Promega), and an ATP-regenerating system (Tatham et al., 2001) were incubated at 37°C for 4–6 hr and analyzed by SDS-PAGE.

### Detection of Endogenous SUMOylated Proteins

Cells were lysed in SDS lysis buffer and sonicated. DTT was added to 10 mM; lysates were diluted to 0.1% SDS, incubated on ice (30 min), precleared, and mixed with anti-Nop58 antibody (Human Protein Atlas; 2 μg/10-cm dish) or ChromPure rabbit IgG (Jackson) and protein G Sepharose (GE; ∼4 hr; 4°C). Bound proteins were washed with 1% NP-40/10 mM IAA/PBS and eluted in SDS-PAGE loading buffer.

### Western Blotting

Primary antibodies: rabbit anti-Nop58 (HPA; 1:1000 or a custom-made antibody to be described elsewhere; 1:250), rabbit anti-SUMO1 (1:500; Santa Cruz), sheep anti-SUMO1 and anti-SUMO2 (gifts from R. Hay; 1:500–1000), rabbit anti-SUMO2 (1:1000; Vertegaal et al., 2004), rabbit anti-SENP3 and anti-SENP5 (gifts from M. Dasso; 1:2000 and 1:1000), mouse anti-B23 and anti-α-tubulin (Sigma-Aldrich; both 1:5000), rabbit anti-lamin B1 (Abcam; 1:1000) and mouse anti-GFP (Roche; 1:1000). HRP-conjugated antibodies (Jackson), and ECL Plus (GE) or Clean-blot IP reagent (Thermo Scientific) were used for secondary detection.

### RNA and Protein Coimmunoprecipitations and Real-Time Quantitative PCR

Cells were harvested, lysed in HNTG buffer and centrifuged (15,000 g, 10 min, 4°C). GFP-TrapA beads (Chromotek) were incubated with cell extract (2 hr; 4°C). The control IP was protein G Sepharose beads coated with anti-HA antibody (3F100, Roche) incubated with U2OS^WTNop58-GFP^ lysates. Beads were washed four times before RNA extraction with Tri-reagent (Invitrogen) and RQ1 DNase treatment (1 hr, 37°C). RNAs (1 μg) were used for a two-step RT-PCR using Superscript II (200U; Invitrogen), N6 random primers (2.5 μM), and dNTPs (0.5 mM). cDNA (10 ng) was used in real-time qPCR using Platinum Taq polymerase (Invitrogen), SYBR Green, and 500 nM specific primers (Supplemental Information and Lutfalla and Uze [2006]) using an Mx3000P system (Stratagene).

## Figures and Tables

**Table 1:** Selected Entries for Putative Nucleolar SUMO1 Substrates from MaxQuant Analysis

Number in Figure 1	Protein Names	Gene Names	Uniprot	ELUATE Seq. Cov. (%)	ELUATE Log2 M/L Norm.	ELUATE M/L Sign. A	ELUATE M/L Count	INPUT Whole M/L Sign. B	SUMO Site?	Other Ref.
1	Alkaline phosphatase; tissue-nonspecific isozyme	ALPL	P05186	15.6	0.768	7.45E-03	3	0.359	No	–

2	H/ACA ribonucleoprotein complex subunit 4	DKC1	A8MUT5	24.5	1.40	3.78E-06	10	0.234	Yes	Manza et al., 2004; Blomster et al., 2009
3	H/ACA ribonucleoprotein complex subunit 2	NHP2	Q9NX24	39.9	3.79	4.42E-34	3	0.150	Yes	Blomster et al., 2009; Golebiowski et al., 2009
4	Nucleolar phosphoprotein p130	NOLC1	Q14978	8.2	2.83	7.41E-20	7	0.358	Yes	Golebiowski et al., 2009
5	Nucleolar protein 58	NOP58	Q9Y2X3	53.7	1.81	3.64E-09	101	0.148	Yes	Zhao et al., 2004; Manza et al., 2004; Schimmel et al., 2008; Golebiowski et al., 2009

6	Alkaline phosphatase; placental type	ALPP	P05187	28.5	1.03	5.24E-04	18	5.84E-09	Yes	Vertegaal et al., 2006

7	Caveolin	CAV1	Q59E85	13.4	0.848	3.54E-03	2	2.81E-04	Yes	–

8	Cytochrome b5 type B	CYB5B	O43169	28.7	0.748	8.87E-03	3	0.136	Yes	–

9	DNA damage-binding protein 2	DDB2	Q92466	44.3	0.909	1.94E-03	20	–	No	Vertegaal et al., 2006; Pungaliya et al., 2007

10	Nucleolar RNA helicase 2	DDX21	Q9NR30	3.1	2.25	3.35E-13	2	0.466	Yes	Matafora et al., 2009

11	Exosome component 10	EXOSC10	Q01780	28.2	3.82	1.05E-34	21	0.374	Yes	Zhao et al., 2004; Golebiowski et al., 2009

12	Four and a half LIM domains 1 variant	FHL1	Q53FI7	43.6	1.30	1.74E-05	14	3.85E-06	No	Wang et al., 2007

13	Lanosterol synthase	LSS	P48449	4.9	0.938	1.44E-03	3	–	No	–

14	Protein arginine N-methyltransferase 5	PRMT5	O14744	7.7	0.805	5.32E-03	3	2.58E-02	Yes	–

15	Retinol dehydrogenase 10	RDH10	Q8IZV5	23.8	1.03	5.27E-04	5	1.22E-03	Yes	–

16	Small ubiquitin-related modifier 1	SUMO1	P63165	46.9	4.55	3.17E-48	37	3.53E-139	No	–

17	Transferrin receptor protein 1	TFRC	P02786	3.9	1.07	3.26E-04	2	5.08E-04	Yes	Golebiowski et al., 2009

18	Transmembrane protein 109	TMEM109	Q9BVC6	9.1	0.805	5.32E-03	3	9.24E-03	No	–

19	Tubulin–tyrosine ligase-like protein 12	TTLL12	Q14166	3.9	0.764	7.66E-03	2	9.71E-02	Yes	Golebiowski et al., 2009

The first entry of each column for protein groups with Ratio M/L Significance A < 0.01 (ratio count >1) is shown. Reverse, contaminant, and unnamed protein groups are not included. “–” indicates a missing value in the MaxQuant output. All columns were derived from Ni^2+^-NTA pull-downs except for “INPUT Whole M/L Significance B,” which is based on analysis of mixed lysates prior to fractionation (Figure 1B). Protein sequences that contain a high probability SUMOylation site are indicated (SUMOplot). snoRNP-related proteins are highlighted. The complete MaxQuant output is given in Tables S1 and S2.

**Table 2:** Selected Entries for Putative Nucleolar SUMO2 Substrates from MaxQuant Analysis

Number in Figure 1	Protein Names	Gene Names	Uniprot	ELUATE Seq. Cov. (%)	ELUATE Log2 H/L Norm.	ELUATE H/L Sign. A	ELUATE H/L Count	INPUT Whole H/L Sign. B	SUMO Site?	Other Ref.
1	Alkaline phosphatase; tissue-nonspecific isozyme	ALPL	P05186	15.6	2.46E	1.04E-03	3	6.94E-21	No	–

2	H/ACA ribonucleoprotein complex subunit 4	DKC1	A8MUT5	24.5	3.34	1.30E-05	10	0.305	Yes	Manza et al., 2004; Blomster et al., 2009
3	H/ACA ribonucleoprotein complex subunit 2	NHP2	Q9NX24	39.9	5.26	1.56E-11	3	0.177	Yes	Blomster et al., 2009; Golebiowski et al., 2009
4	Nucleolar phosphoprotein p130	NOLC1	Q14978	8.2	2.86	1.66E-04	7	0.292	Yes	Golebiowski et al., 2009
5	Nucleolar protein 58	NOP58	Q9Y2X3	53.7	3.61	2.68E-06	101	3.15E-02	Yes	Zhao et al., 2004; Manza et al., 2004; Schimmel et al., 2008; Golebiowski et al., 2009

20	Cyclin-dependent kinase inhibitor 2A; isoform 4	CDKN2A	Q8N726	39.9	5.29	1.20E-11	6	5.67E-18	No	–

21	Glucose-6-phosphate isomerase	GPI	P06744	45.9	2.24	2.59E-03	36	0.497	Yes	Manza et al., 2004

22	KLHDC3 protein	KLHDC3	Q96GH7	22.9	2.14	3.82E-03	5	–	No	–

23	Nuclear factor 1 X-type	NFIX	Q14938	10.2	2.30	2.00E-03	2	–	Yes	Golebiowski et al., 2009

24	PDZ and LIM domain protein 7	PDLIM7	Q9NR12	23.9	2.24	2.54E-03	12	1.77E-06	Yes	–

25	Small ubiquitin-related modifier 3	SUMO3	A8MU27	17	5.70	2.93E-13	18	1.16E-07	Yes	–

Same as Table 1 except that the first entry of each column for protein groups with ratio H/L significance A < 0.01 (with ratio count >1) is shown.

## Supplemental Information

Document S1. Five Figures, Two Tables, Supplemental Experimental Procedures, and Supplemental References

Table S1 Related to Tables 1 and 2 and Figure 1. Excel spreadsheet of “protein groups” file generated by MaxQuant analysis of eluates following denaturing Ni^2+^-NTA pull-downs of SILAC-labeled lysates to identify nucleolar SUMO targets.

Table S2 Related to Tables 1 and 2 and Figure 1. Excel spreadsheet of “protein groups” file generated by MaxQuant analysis of “inputs,” i.e., mixed SILAC-labeled lysates prior to fractionation and denaturing Ni^2+^-NTA pull-downs.
