# Supplementary material for: A Proteomic Screen for Nucleolar SUMO Targets Shows SUMOylation Modulates the Function of Nop5/Nop58
Source: Mol Cell. 2010 Aug 27;39(4):618–31. doi: 10.1016/j.molcel.2010.07.025 (PMC2938476; doi:10.1016/j.molcel.2010.07.025)
Supplement: Document S1. Five Figures, Two Tables, Supplemental Experimental Procedures, and Supplemental References [file mmc1.pdf]

Molecular Cell, Volume *39*

Supplemental Information

A Proteomic Screen for Nucleolar SUMO Targets Shows SUMOylation Modulates the Function of Nop5/Nop58

Belinda Jane Westman, Céline Verheggen, Saskia Hutten, Yun Wah Lam, Edouard Bertrand, and Angus Lamond

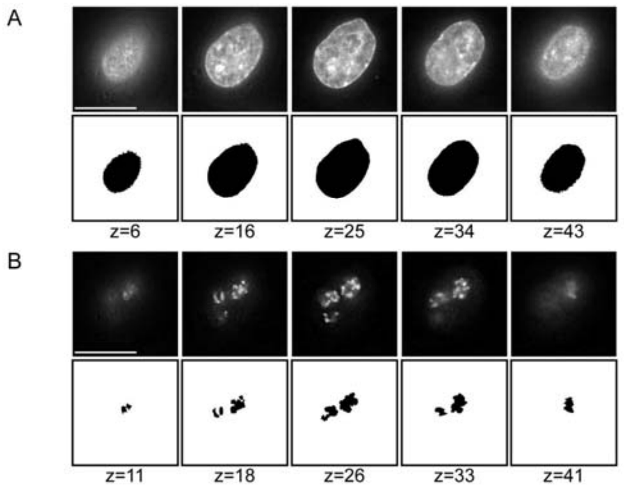

Figure S1

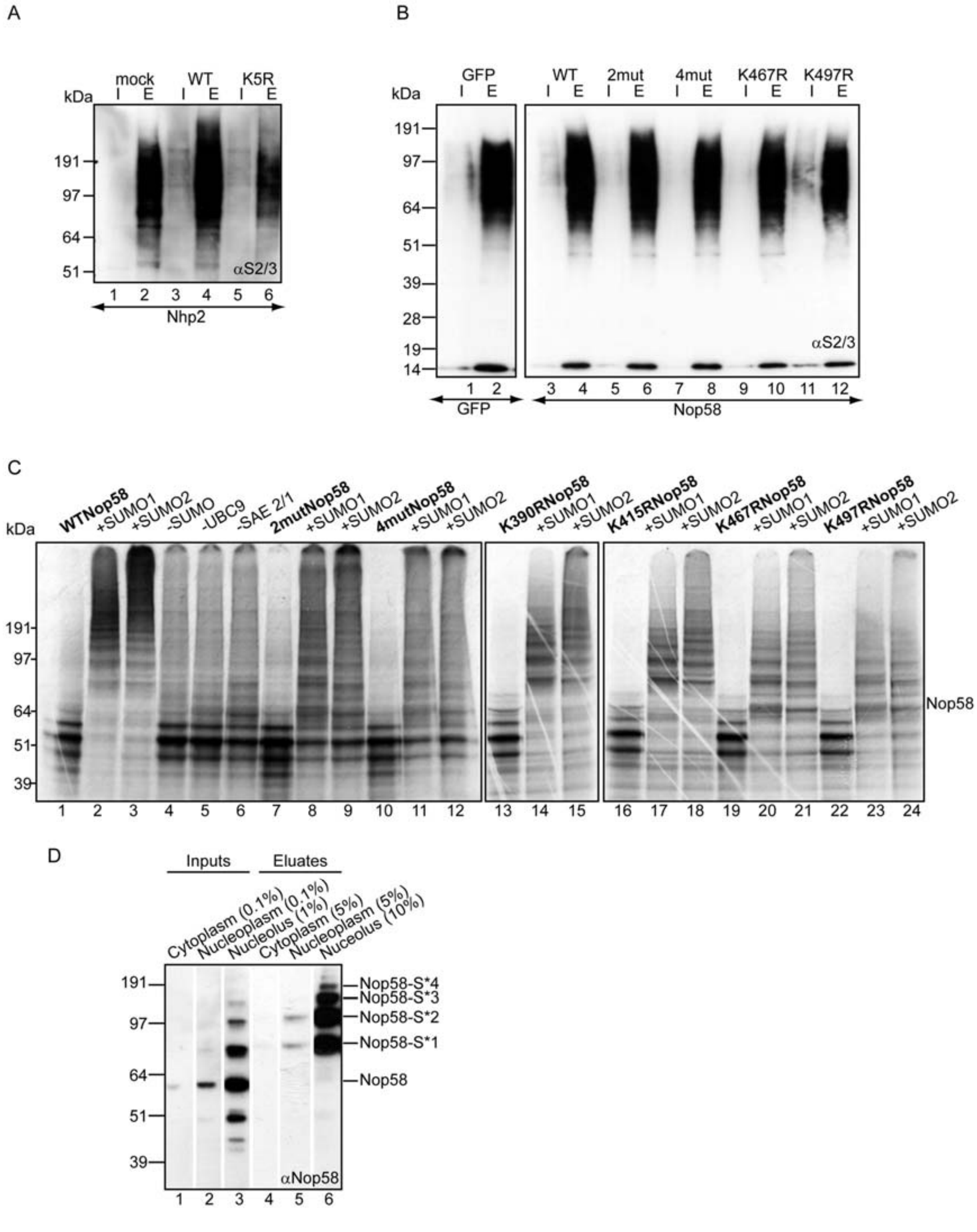
A
kDa
mock
WT
K5R
I E I E I E
191
97
64
51
αS2/3
1 2 3 4 5 6
Nhp2
B
kDa
GFP
WT
2mut
4mut
K467R
K497R
I E I E I E I E I E I E
191
97
64
51
39
28
19
14
αS2/3
1 2 3 4 5 6 7 8 9 10 11 12
GFP
Nop58
C
kDa
WTNop58
+SUMO1
+SUMO2
-SUMO
-UBC9
-SAE 2/1
2mutNop58
+SUMO1
+SUMO2
4mutNop58
+SUMO1
+SUMO2
K390RNop58
+SUMO1
+SUMO2
K415RNop58
+SUMO1
+SUMO2
K467RNop58
+SUMO1
+SUMO2
K497RNop58
+SUMO1
+SUMO2
191
97
64
51
39
Nop58
1 2 3 4 5 6 7 8 9 10 11 12 13 14 15 16 17 18 19 20 21 22 23 24
D
Inputs
Eluates
Cytoplasm (0.1%)
Nucleoplasm (0.1%)
Nucleolus (1%)
Cytoplasm (5%)
Nucleoplasm (5%)
Nuceolus (10%)
191
97
64
51
39
Nop58-S*4
Nop58-S*3
Nop58-S*2
Nop58-S*1
Nop58
αNop58
1 2 3 4 5 6

Figure S2

A

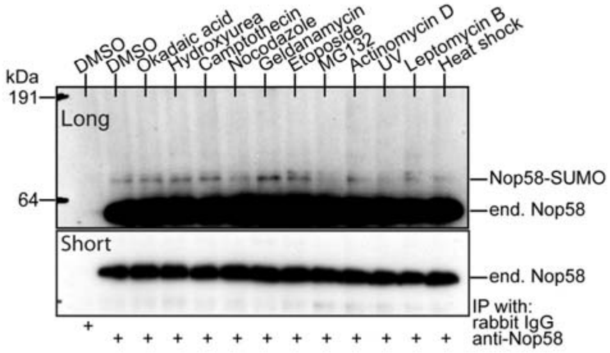
kDa
191
64
DMSO
DMSO
Okadaic acid
Hydroxyurea
Camptothecin
Nocodazole
Geldanamycin
Etoposide
MG132
Actinomycin D
UV
Leptomycin B
Heat shock
Long
Nop58-SUMO
end. Nop58
Short
end. Nop58
IP with:
rabbit IgG
anti-Nop58

Figure S3

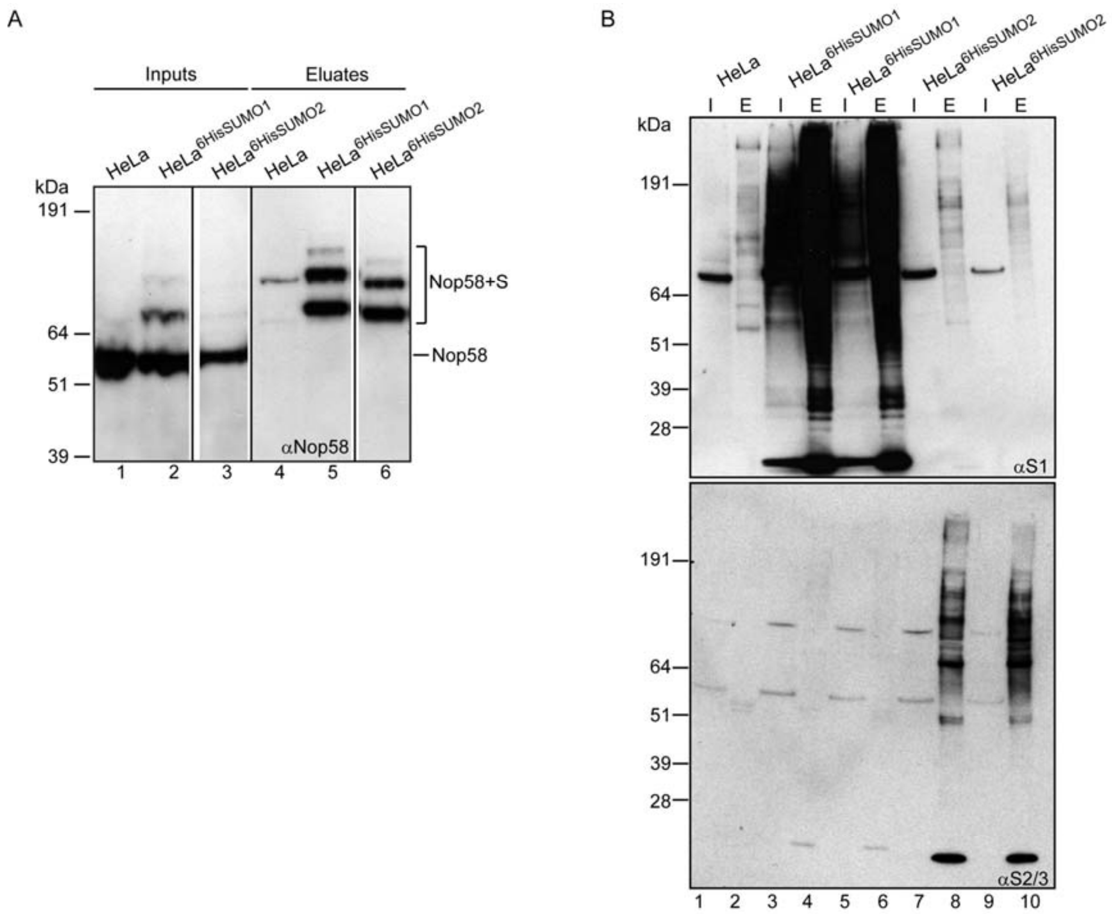

A
Inputs
Eluates
HeLa
HeLa6HisSUMO1
HeLa6HisSUMO2
kDa
191
64
51
39
Nop58+S
Nop58
αNop58
1 2 3 4 5 6
B
I E I E I E I E I E
191
64
51
39
28
αS1
αS2/3
1 2 3 4 5 6 7 8 9 10

Figure S4

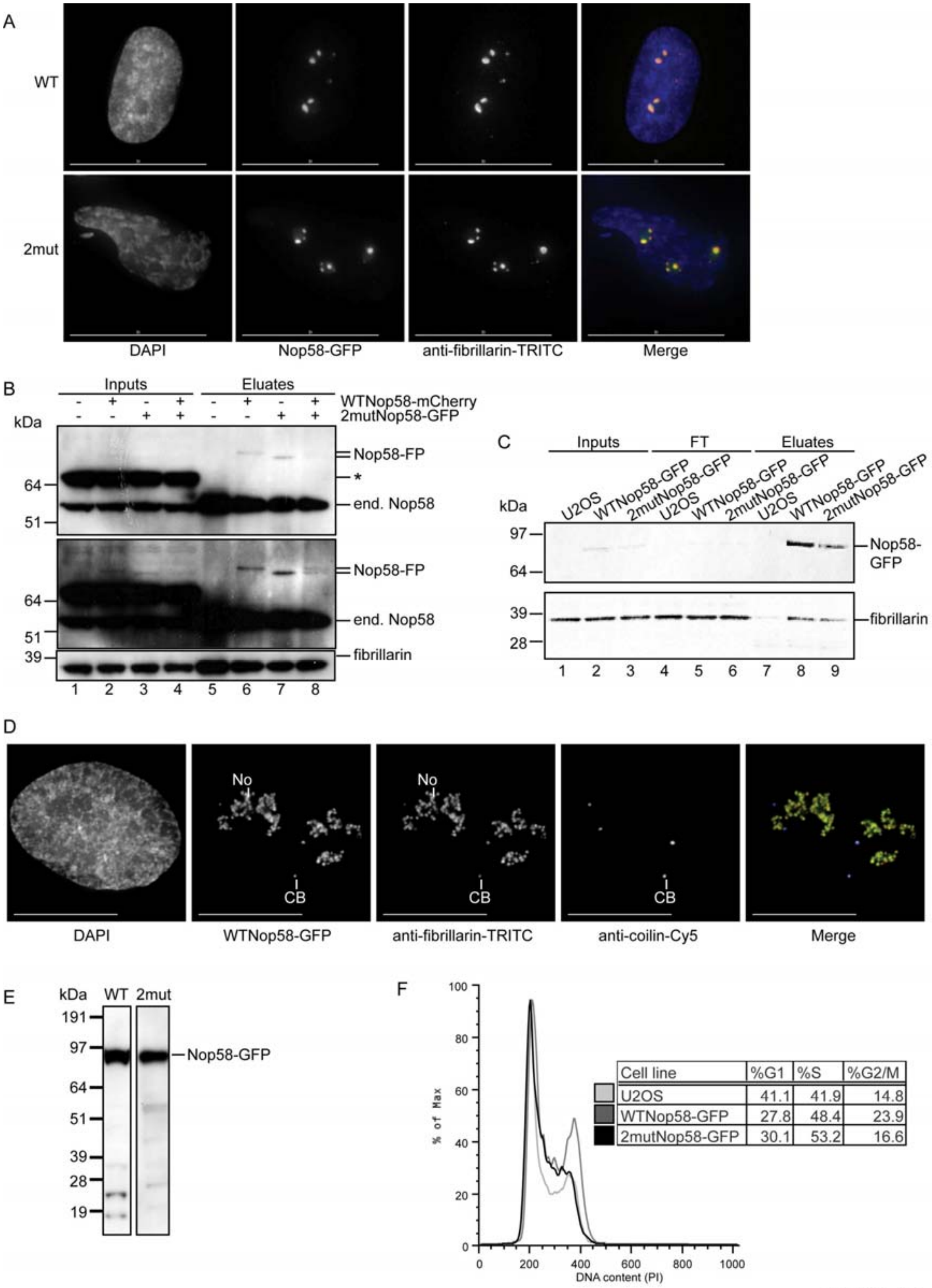
A
WT
2mut
DAPI
Nop58-GFP
anti-fibrillarin-TRITC
Merge
B
Inputs
Eluates
WTNop58-mCherry
2mutNop58-GFP
kDa
Nop58-FP
*
end. Nop58
fibrillarin
64
51
39
1 2 3 4 5 6 7 8
C
Inputs
FT
Eluates
U2OS
WTNop58-GFP
2mutNop58-GFP
kDa
97
64
39
28
Nop58-GFP
fibrillarin
1 2 3 4 5 6 7 8 9
D
No
CB
DAPI
WTNop58-GFP
anti-fibrillarin-TRITC
anti-coilin-Cy5
Merge
E
kDa
WT
2mut
191
97
64
51
39
28
19
Nop58-GFP
F
% of Max
DNA content (PI)
Cell line | %G1 | %S | %G2/M
U2OS | 41.1 | 41.9 | 14.8
WTNop58-GFP | 27.8 | 48.4 | 23.9
2mutNop58-GFP | 30.1 | 53.2 | 16.6

Figure S5

Figure S1. Example of the use of connected threshold connectivity to accurately define nuclear and nucleolar regions in Image J to allow the amount of nucleolar vs nuclear SUMO to be estimated. Related to Fig. 1.

Nuclear and nucleolar regions were defined based on the intensities of the staining patterns of either Hoechst (A) or anti-fibrillarin antibodies (B), respectively, as detailed in the Exp. Proc. A selection of IF images across the z-stack for a single cell is shown, along with the corresponding nuclear or nucleolar regions (black) as defined using our semi-automated Image J plug-in. Intensities resulting from staining with anti-SUMO antibodies were then summed for pixels that belonged to either or both regions and used to calculate the average percentage of nucleolar SUMO1 or 2/3.

Figure S2. Identification of SUMOylation sites in Nhp2 and Nop58. Related to Fig. 2.

(A, B) Blots shown in Figure 2B and C (lanes 1–12) were stripped and reprobed with antibodies against SUMO2 to confirm that the Ni^{2+}-NTA pulldowns had successfully enriched SUMO2-modified proteins.

(C) *In vitro* expressed and ^{35}S-Met-labeled Nop58, 2mutNop58 (K467R, K497R), 4mutNop58 (K390R, K415R, K467R, K497R), K390R-Nop58, K415R-Nop58, K467R-Nop58 and K497R-Nop58 and were incubated with ATP, recombinant SUMO1, SUMO2 or GST-SUMO2, Ubc9 and SAE 2/1 as indicated. Reaction products were fractionated by SDS-PAGE and the dried gel was exposed to X-ray film for visualisation. The position of unmodified Nop58 is indicated. Modification of 2mut-, 4mut-, K467R- and K497R-Nop58 was less efficient than that of WTNop58, as indicated by the decrease of slower migrating bands and increased level of unmodified Nop58 (lanes 8, 9, 11, 12, 20, 21, 23 and 24).

(D) The majority of SUMOylated Nop58 localises to the nucleolus. Cytoplasmic, nucleoplasmic and nucleolar fractions from $HeLa^{6HisSUMO1}$ cells were subjected to denaturing Ni^{2+}-NTA pulldowns, and input and eluate samples analysed by Western blotting using an anti-Nop58 antibody. The percentage of each sample that was loaded onto the gel is indicated.

Figure S3. Nop58 SUMOylation does not increase after stress. Related to Fig. 3.

(A) U2OS cells were subjected to a variety of treatments and Nop58 SUMOylation analysed by IP of endogenous Nop58 with either control or anti-Nop58 antibodies, followed by Western blotting analysis of input (not shown) and eluted proteins. A long and a short exposure of the ECL reaction are shown. The positions of Nop58 and Nop58 attached to a single SUMO molecule (Nop58-SUMO) are indicated.

Figure S4. Nop56 and 15.5K are poor SUMO substrates. Related to Fig. 4.

(A) Blot shown in Fig. 4E was stripped and reprobed with antibodies against Nop58 to confirm that the Ni^{2+}-NTA pulldowns had successfully enriched SUMO-modified proteins.

(B) Blot shown in Fig. 4F was stripped and reprobed with antibodies against either SUMO1 (top) or SUMO2/3 (bottom) to confirm that the Ni^{2+}-NTA pulldowns had successfully enriched SUMO1 or SUMO2-modified proteins.

Figure S5. Towards the function of Nop58 SUMOylation. Related to Fig. 5.

(A) Nop58 SUMOylation is not required for the sub-cellular localisation of Nop58 after treatment with actinomycin D. $U2OS^{WTNop58\text{-}GFP}$ and $U2OS^{2mutNop58\text{-}GFP}$ cells (as indicated) were treated with actinomycin D (1 μg/ml, 3h), fixed and co-stained with DAPI and anti-fibrillarin antibodies. Images recorded in each channel are shown separately as greyscale images, whereas the merged image using the blue (DAPI), green (Nop58-GFP) and red (fibrillarin) channels is shown in colour. Each image is a 2D projection of the entire nuclear fluorescence and scale bars represent 30 μm.

(B, C) Nop58 SUMOylation is required but not essential for snoRNP formation. (B) U2OS cells were transfected for 48 h with WTNop58-mCherry and/or 2mutNop58-GFP, and endogenous fibrillarin was IP'd. Control IPs were carried out using mock-transfected cells. Western blots were performed with anti-Nop58 (top and middle; short and long exposures, respectively) and anti-fibrillarin (bottom) antibodies. The Nop58-FPs are less abundant than endogenous Nop58 but are enriched after anti-fibrillarin IPs (eluates; lanes 6–8). The intensities of the bands corresponding to either WTNop58-mCherry, or 2mutNop58-GFP, are almost identical in the double transfection (lane 8), suggesting that

both proteins have similar affinities for fibrillarin and are incorporated at similar levels in box C/D snoRNPs. Similar amounts of fibrillarin were isolated in each IP (bottom; lanes 5–8). The positions of Nop58, Nop58-FP and fibrillarin are indicated. “*” = non-specific background band. (C) WTNop58- and 2mutNop58-GFP were IP’d from nuclear extracts of U2OS$^{WTNop58\text{-}GFP}$ and U2OS$^{2mutNop58\text{-}GFP}$ cells using anti-GFP beads. U2OS cells were used as a control. Input, unbound (flow-through) and eluated proteins were analysed by quantitative Western blotting using anti-GFP (top) and anti-fibrillarin (bottom) antibodies. The normalised ratios of the intensities of Nop58-GFP: fibrillarin bands are 1 and 0.6 for WT and 2mut (lanes 8 and 9), respectively.

(D, E, F) Characterisation of the U2OS$^{WTNop58\text{-}GFP}$ and U2OS$^{2mutNop58\text{-}GFP}$ stable cell lines used to investigate the association of Nop58 with snoRNAs and fibrillarin. Fixed U2OS$^{WTNop58\text{-}GFP}$ and U2OS$^{2mutNop58\text{-}GFP}$ cells (not shown) were examined by fluorescence microscopy, and the co-localisation of each FP with both nucleoli (No) and Cajal bodies (CB) was revealed by co-staining cells as indicated. Images recorded in each channel are shown separately as greyscale images, whereas the merged image using the green (WTNop58-GFP), red (fibrillarin) and far-red (coilin) channels is shown in colour. Each image is a 2D projection of the entire nuclear fluorescence and scale bars represent 15 μm. In (E), total cell lysates of U2OS$^{WTNop58\text{-}GFP}$ and U2OS$^{2mutNop58\text{-}GFP}$ cells were analysed by SDS-PAGE and Western blotting with anti-GFP antibodies to confirm that each FP migrated as a single band at the expected molecular weight. In (F), U2OS$^{WTNop58\text{-}GFP}$ and U2OS$^{2mutNop58\text{-}GFP}$ cells were stained with propidium iodide (PI) and their DNA content examined by flow cytometry to reveal that stable expression of each FP in U2OS cells has only a minor impact on cell cycle progression.

Supplemental Experimental Procedures

Plasmids made for this study

All plasmid sequences were verified by sequencing.

**Insert**	**Vectors used (promoter for in vitro transcription)**
Nop58	pEGFPN1, pcDNA3.1 (T7), mCherryN1
2mutNop58	pEGFPN3, pcDNA3.1 (T7)
4mutNop58	pEGFPN3, pcDNA3.1 (T7)
K390R-Nop58	pEGFPN3, pcDNA3.1 (T7)
K415R-Nop58	pEGFPN3, pcDNA3.1 (T7)
K467R-Nop58	pEGFPN3, pcDNA3.1 (T7)
K497R-Nop58	pEGFPN3, pcDNA3.1 (T7)
EEAA-Nop58	pEGFPN1
Nhp2	pEGFPN1, pcDNA3.1 (T7)
Nop56	pcDNA3.1 (T7)
6HisSUMO1	pcDNA3.1
6HisSUMO2	pcDNA3.1
6HisSUMO3	pcDNA3.1
IRF2	pcDNA3.1 (T7)
15.5K	pcDNA3.1 (T7)

Generation of stable cell lines

To generate U2OS$^{WTNop58\text{-}GFP}$ and U2OS$^{2mutNop58\text{-}GFP}$ lines, cells were put under selection 24 h following transfection (G418; 400 µg/ml). After 2 wks of selection, resistant colonies were picked, propagated and characterised.

Buffers

PHEM buffer: 30 mM HEPES, 65 mM PIPES pH 6.9, 10 mM EGTA, 2 mM $MgCl_2$

SDS lysis buffer: 1% SDS, 1% NP-40, 10 mM IAA in PBS supplemented with Complete protease inhibitor cocktail, EDTA-free (Roche)

HNTG buffer: 20 mM HEPES pH 7.9, 150 mM NaCl, 1 mM $MgCl_2$, 1 mM EGTA, 10 % glycerol, 1 % Triton-X100 containing Complete protease inhibitor cocktail, EDTA-free (Roche)

Sonication buffer: 40 mM Tris pH 7.5, 200 mM NaCl, 0.05% NP-40, supplemented with Complete protease inhibitor cocktail, EDTA-free (Roche)

U2OS Buffer A: 20 mM Tris pH 7.4, 10 mM KCl, 3 mM $MgCl_2$, 0.1% NP-40, 10% glycerol with Complete protease inhibitor cocktail, EDTA-free (Roche)

RIPA buffer: 50 mM Tris pH 7.4, 150 mM NaCl, 1% NP-40, 0.5% sodium deoxycholate with Complete protease inhibitor cocktail, EDTA-free (Roche)

siRNA sequences (Thermo Scientific) and concentrations

<table>
<tr><th>Name</th><th>Sequence</th><th>Concentration for knockdown I</th><th>Concentration for knockdown II</th></tr>
<tr><td>SENP3.1</td><td>ACGAAUUCCUUCAAACGUA</td><td rowspan="4">10 nM</td><td rowspan="8">10 nM</td></tr>
<tr><td>SENP3.2</td><td>GCACUGAUGAGGUAGUAGA</td></tr>
<tr><td>SENP3.3</td><td>GAUAAACUCCGUACCAAGG</td></tr>
<tr><td>SENP3.4</td><td>CAAGUCAGGUGGAGGGUUU</td></tr>
<tr><td>SENP5.1</td><td>GUACAGAGCUGAUUCAUGA</td><td rowspan="4">10 nM</td></tr>
<tr><td>SENP5.2</td><td>GAGGAAAGGAAUCCACUUA</td></tr>
<tr><td>SENP5.3</td><td>UAACCAACAUAGAAGGAUA</td></tr>
<tr><td>SENP5.4</td><td>GGGCAAGGAGCUUAGUUUA</td></tr>
<tr><td>Nop58 3’UTR</td><td>GGGAAGGTTCAGTAAGACA</td><td rowspan="2">75 nM</td><td rowspan="2">-</td></tr>
<tr><td>Nop58 5’UTR</td><td>TGAACTGACTCTACAGCTT</td></tr>
<tr><td>FFL (Firefly luciferase)</td><td>CUUACGCUGAGUACUUCGA3</td><td>75 nM</td><td>-</td></tr>
</table>

Image-J plug-in for quantitation of nucleolar fluorescence

For quantitation of nucleolar fluorescence, deconvolved images (SoftWorx) were parsed as split channels by the Bio-formats Importer plug-in (http://www.loci.wisc.edu/ome/formats-imagej.html) into the Image J software package (http://rsb.info.nih.gov/ij/index.html) using the auto-scale function all channels. Cell nuclei were selected as the regions of interest (ROI). Briefly, pixels were defined as

belonging to these regions if they displayed intensities greater than a "high" threshold, or if they displayed intensities greater than a "low" threshold and were adjacent to another pixel that had also been defined as either nuclear or nucleolar. The same thresholds were applied across the entire z-stack for each cell. This plug-in also enabled the mapping of pixel intensities resulting from the anti-SUMO staining to the pixel location, *i.e.*, nuclear or nucleolar, across the entire z-stack.

Immunofluorescence

Cells were fixed and stained according to the Exp. Proc., with the following antibodies: mouse anti-fibrillarin (72B9; 1 in 10; (Reimer *et al.*, 1987)), rabbit anti-coilin (204/10; 1 in 300; (Bohmann *et al.*, 1995)). Images were acquired and processed according to Exp. Proc.

Preparation of nucleolar extracts and purification of 6HisSUMO-conjugated proteins to examine 6HisSUMO-Nop58 localisation

Cytoplasmic, nucleoplasmic and nucleolar fractions were prepared from HeLa^{6HisSUMO1} cells as described previously (Andersen *et al.*, 2002). Cytoplasmic and nucleoplasmic fractions were diluted ten-fold with 6M Gdn-HCl-containing lysis buffer. Purified nucleoli were solubilised in 6M Gdn-HCl-containing lysis buffer. 6HisSUMO-conjugated proteins were purified using Ni^{2+}-NTA agarose (Qiagen) as in the main text (Tatham *et al.*, 2009).

Immunoprecipitation of fibrillarin-containing complexes

Endogenous fibrillarin was IP'd from HeLa cells lysed in sonication buffer. Anti-fibrillarin 72B9 (5 μg; Cytoskeleton) was incubated with Protein-G Sepharose (~100 μg; GE) for 1 h in SB. Unbound antibody was removed by washing with SB and antibody-coated beads were added to pre-cleared lysates for IPs (3 h, 4 °C). Bound proteins were washed with SB.

To IP WTNop58- and 2mutNop58-GFP from U2OS$^{WTNop58\text{-}GFP}$ and U2OS$^{2mutNop58\text{-}GFP}$ cell nuclei, pellets were resuspended in U2OS Buffer A, incubated (10 min; ice) and spun (2,000 rpm, 5 min; 4 °C). Supernatant was retained as the cytoplasmic fraction. Nuclear pellets were washed with U2OS Buffer A, resuspended in RIPA buffer, sonicated, spun

(13,000 rpm, 10 min, 4 °C) and pre-cleared with Protein G-Sepharose beads. Extracts were incubated with GFP-TrapA beads (Chromotek, Germany) for 4 h (4 °C). Beads were washed (4 x 1 ml of RIPA buffer).

In both cases, bound proteins were eluted with SDS-PAGE loading buffer, and analysed by Western blotting using the following primary antibodies: mouse anti-GFP (Roche; 1 in 1000), rabbit anti-fibrillarin serum 42 (gift from F. Fuller-Pace) or anti-Nop58 (main text). For Fig. S5C, the secondary antibodies used were anti-mouse AF680 (Invitrogen) and anti-rabbit IRDye800 (Rockland) and proteins were visualised and quantitated on an Odyssey Imager (Li-Cor Biosciences).

Cell cycle analysis and flow cytometry

To detect the percentage of GFP-positive cells in $U2OS^{WTNop58\text{-}GFP}$ and $U2OS^{2mutNop58\text{-}GFP}$ lines, samples were prepared by harvesting cells with trypsin and resuspending in PBS/1% FBS. For cell cycle analysis, cells were trypsinised and then fixed in 70% ethanol for 30 min at RT and stained with propidium iodide (50 μg/ml) containing RNAse-A (50 μg/ml) and 0.1% Triton X-100. Data were acquired on a FACS Calibur cytometer (Becton Dickinson, USA) using CellQuest software and analysed using FlowJo (Treestar, USA) software. Viable cells were gated according to their forward scatter and side scatter profiles.

Primers used for real-time qPCR

Thermal cycling parameters were 10 min at 95 °C, followed by 40 cycles at 95 °C for 30 s, 62 °C for 30 s and 72 °C for 1 min.

Target RNA	Forward primer	Reverse primer
U2	TCGGCCTTTTGGCTAAGATC	CCTGGAGGTACTGCAATACC
GAPDH	ACACCCACTCCTCCACCTTT	TCCACCACCCTGTTGCTGTA
U3	CCACGAGGAAGAGAAGTAGCG	ACCACTCAGACCGCGTTCTCAT
U8	TCCGGAGGGCAGATTAGAACATGA	GGATGTTGCAAGACCTGATTACGC
U13	GTTCATGAGCGTGATGATTGGGTG	ACATGGTAACAAGGTTCGAGGGTG
U14	CTCAGTAAATGTTAGCTGTGG	TACATCACAGAAACCAACGTG

Drug treatments

Drug	Final concentration in media	Time
Okadaic acid	10 nM	24 h
Hydroxy urea	2 mM	24 h
Camptothecin	25 nM	24 h
Nocodazole	100 ng/ml	16 h
Geldanamycin	7 μM	16 h
Etoposide	10 μM	10 min with drug and harvest cells after 6 h
MG132	5 μM	5 h
UV	NA	Expose cells to UV (30 J/m^2) then harvest after 3 h
Actinomycin D	1 μg/ml	3 h
Leptomycin B	10 nM	3 h
Heat shock	NA	30 min at 42 °C

Supplemental References

Andersen, J.S., Lyon, C.E., Fox, A.H., Leung, A.K., Lam, Y.W., Steen, H., Mann, M., and Lamond, A.I. (2002). Directed proteomic analysis of the human nucleolus. Curr. Biol. *12*, 1-11.

Bohmann, K., Ferreira, J.A., and Lamond, A.I. (1995). Mutational analysis of p80 coilin indicates a functional interaction between coiled bodies and the nucleolus. J. Cell Biol. *131*, 817-831.

Reimer, G., Pollard, K.M., Penning, C.A., Ochs, R.L., Lischwe, M.A., Busch, H., and Tan, E.M. (1987). Monoclonal autoantibody from a (New Zealand black x New Zealand white)F1 mouse and some human scleroderma sera target an Mr 34,000 nucleolar protein of the U3 RNP particle. Arthritis. Rheum. *30*, 793-800.

Tatham, M.H., Rodriguez, M.S., Xirodimas, D.P., and Hay, R.T. (2009). Detection of protein SUMOylation in vivo. Nat Protoc *4*, 1363-1371.
